# Mothers’ Attachment Representations and Children’s Brain Structure

**DOI:** 10.3389/fnhum.2022.740195

**Published:** 2022-03-16

**Authors:** Megan H. Fitter, Jessica A. Stern, Martha D. Straske, Tamara Allard, Jude Cassidy, Tracy Riggins

**Affiliations:** ^1^Maryland Child and Family Development Lab, University of Maryland, College Park, College Park, MD, United States; ^2^BabyLab, University of Virginia, Charlottesville, VA, United States; ^3^Neurocognitive Development Lab, University of Maryland, College Park, College Park, MD, United States

**Keywords:** attachment, secure base scripts, amygdala, hippocampus, parenting, early childhood, brain structure

## Abstract

Ample research demonstrates that parents’ experience-based mental representations of attachment—cognitive models of close relationships—relate to their children’s social-emotional development. However, no research to date has examined how parents’ attachment representations relate to another crucial domain of children’s development: *brain development.* The present study is the first to integrate the separate literatures on attachment and developmental social cognitive neuroscience to examine the link between mothers’ attachment representations and 3- to 8-year-old children’s brain structure. We hypothesized that mothers’ attachment representations would relate to individual differences in children’s brain structures involved in stress regulation—specifically, amygdala and hippocampal volumes—in part via mothers’ responses to children’s distress. We assessed 52 mothers’ attachment representations (secure base script knowledge on the Attachment Script Assessment and self-reported attachment avoidance and anxiety on the Experiences in Close Relationships scale) and children’s brain structure. Mothers’ secure base script knowledge was significantly related to children’s smaller left amygdala volume but was unrelated to hippocampal volume; we found no indirect links via maternal responses to children’s distress. Exploratory analyses showed associations between mothers’ attachment representations and white matter and thalamus volumes. Together, these preliminary results suggest that mothers’ attachment representations may be linked to the development of children’s neural circuitry related to stress regulation.

## Introduction

Integral to attachment theory is the claim that individuals form experience-based mental representations of attachment—cognitive models of the nature of close relationships (Bowlby, 1969/1982). Those mental representations of attachment have consequences not only for individuals’ own development, but also for the development of their children (Steele et al., 2014; Jones et al., 2015). The vast majority of research on relations between caregivers’ attachment representations and children’s developmental outcomes examines how parents’ representations link to caregiving behaviors (Coppola et al., 2006; Jones et al., 2015) and their children’s attachment security (e.g., Steele et al., 2014). However, no research to date has examined how caregivers’ attachment representations relate to another crucial domain of child development: *children’s brain development.*

Some research indirectly indicates the possibility of a link between mothers’ attachment representations and children’s brain development. For example, abundant research demonstrates that mothers’ representations relate to children’s attachment security (e.g., van IJzendoorn, 1995; Steele et al., 2014), and an emerging body of research indicates that children’s attachment security is related to individual differences in brain structure (see Long et al., 2020, for a review; see Puhlmann et al., 2021, for evidence in adolescence, see also Ilyka et al., 2021). Furthermore, in addition to the large body of research demonstrating that mothers’ attachment representations guide caregiving behavior (e.g., Huth-Bocks et al., 2014; Jones et al., 2015), some research demonstrates that normative variation in caregiving relates to individual differences in children’s cortical and subcortical brain structures (Kok et al., 2015; Rifkin-Graboi et al., 2015; Farber et al., 2020; Ilyka et al., 2021; Richmond et al., 2021). Thus, although there is some indication of a possible indirect link between mothers’ attachment representations and children’s brain development, no research to date has examined the direct link or mechanisms through which this relation might occur.

The present study is the first to examine whether mothers’ attachment representations relate to their children’s brain structure and whether caregiving explains this link. We focus our examination of caregiving behaviors on *mothers’ responses to children’s distress* because such responses are a key form of co-regulation—external regulation of a child’s physiological rhythms and emotions by a sensitive caregiver (Spangler et al., 1994). Effective co-regulation is thought to support a child’s ability to self-regulate their own physiological and emotional responses to threat (Cassidy, 1994; Hofer, 1995; Luecken and Lemery, 2004; George and Solomon, 2008). For this reason, we focus our examination of children’s brain structure on two principal regions involved in circuits that facilitate stress regulation in times of threat: the hippocampus and the amygdala (McEwen, 2000; Callaghan and Tottenham, 2016a). Our focus on these two brain structures (in addition to their role in stress regulation) stems from an emerging body of evidence that normative variations in attachment security (e.g., Quirin et al., 2010; Lyons-Ruth et al., 2016) and caregiving (e.g., Rifkin-Graboi et al., 2015; Luby et al., 2016) relate to individual differences in both of these brain structures.

We begin with a discussion of attachment representations. Next, in order to support the expectations of a relation between mothers’ representations and children’s brain structure, we discuss the abundance of evidence that mothers’ attachment representations relate to their caregiving. Then, we discuss evidence that mothers’ caregiving relates to children’s brain structure and present the current study.

Mental representations of attachment are experience-based cognitive models that encompass individuals’ beliefs about the self and others—including expectations about how they themselves will be treated in close relationships (Bowlby, 1969/1982; Bretherton and Munholland, 2016). When individuals experience sensitive care from caregivers and other close relationship partners, they develop secure attachment representations. People with secure attachment representations hold beliefs that others will be available and sensitively responsive in times of need, that they themselves are worthy of such care, and that they are capable of and effective in eliciting care when needed. In contrast, experiences of rejection or neglect in close relationships can result in insecure mental representations, characterized by mistrust of others and negative beliefs about the self (Bowlby, 1969/1982).

There are multiple ways that researchers operationalize attachment representations. Two widely used constructs are *secure base script knowledge* and *attachment style*. The secure base script is a knowledge structure, or schema, consisting of a sequence of events wherein a person in distress seeks and receives care from a close other, and then the distress is resolved as a function of that care (Bretherton, 1985, 1987; Waters and Waters, 2006). The extent to which a person organizes information following this schema reflects their secure base script knowledge. The other widely used conceptualization of attachment representations is attachment style, consisting of two dimensions: attachment avoidance and attachment anxiety (Brennan et al., 1998). *Attachment* a*voidance* reflects the extent to which an individual is uncomfortable with intimacy and with depending on relationship partners for support. *Attachment anxiety* reflects the extent to which an individual is preoccupied with relationships and fears rejection and abandonment by relationship partners. High levels of attachment anxiety, attachment avoidance, or both indicate an insecure attachment style. Low levels of both anxiety and avoidance indicate attachment security, characterized by feelings that one is worthy of love and care, and that relationship partners will be available and responsive in times of need (Mikulincer and Shaver, 2007).

Although secure base script knowledge and attachment style capture subtly different aspects of mental representations, the two are closely intertwined. Secure base scripts are considered to be the raw building blocks of more complex mental representations (Bretherton, 1991). According to theory (Mikulincer and Shaver, 2007), a person who has access to a secure base script is unlikely to fear either rejection and abandonment (fears that are central to anxious attachment styles) or relying on relationship partners for support (fears central to avoidant attachment styles). Secure base script knowledge mitigates these fears because such scripts are characterized by a schema of consistently responsive care and alleviation of distress as a function of that care. As such, secure base scripts lay the foundation for an individual’s secure attachment style. Indeed, empirical data suggest that individuals with a secure attachment style demonstrate high secure base script knowledge (Dykas et al., 2006; Mikulincer et al., 2009).

Mothers’ attachment representations (both secure base script knowledge and attachment style) relate to the quality of care they provide to their children. Ample research reveals that mothers with greater access to secure base script knowledge provide more sensitive care to their children (e.g., Coppola et al., 2006; Huth-Bocks et al., 2014; Waters et al., 2017). For instance, one study demonstrated that mothers’ secure base script knowledge predicted greater positive parenting behaviors (e.g., sensitivity and positive affect) and fewer negative parenting behaviors (e.g., hostility and intrusiveness) during a free play task in the home (Huth-Bocks et al., 2014).

A separate body of evidence reveals links between parents’ insecure attachment style dimensions and a wide array of negative parenting behaviors (see Jones et al., 2015, for a review). Empirical work consistently demonstrates that attachment avoidance relates to low sensitivity and responsiveness (Rholes et al., 1995; Mills-Koonce et al., 2011), whereas links between attachment anxiety and sensitivity are more mixed (Jones et al., 2015). Nevertheless, both anxiety and avoidance are associated with mothers engaging in more frequent and harsh conflict with their children (e.g., Feeney, 2006; Selcuk et al., 2010; La Valley and Guerrero, 2012).

Examination of maternal caregiving is critical because early caregiving experiences become biologically embedded in the child and can have profound effects on development (McEwen, 2000; Meaney and Szyf, 2005; Belsky and de Haan, 2011; McLaughlin et al., 2019). Early experiences with caregivers predict the development of subcortical (e.g., Meaney and Szyf, 2005; Rifkin-Graboi et al., 2015; Gee, 2016; Sethna et al., 2017; Bernier et al., 2019) and cortical (e.g., Blaze et al., 2013; Kok et al., 2015) brain structures. Although abundant research demonstrates that early maltreatment and neglect relate to children’s brain development (see Belsky and de Haan, 2011; McLaughlin et al., 2019, for reviews), we focus here on normative variation in caregiving behaviors.

The hippocampus is sensitive to early stressful caregiving environments; elevated levels of stress hormones (e.g., cortisol) have particularly deleterious consequences for regions with an abundance of glucocorticoid receptors, such as the hippocampus (Sapolsky et al., 1986; McEwen, 2000; Conrad, 2009). As such, if caregivers cannot effectively regulate a child’s distress, then it is possible that the child may experience alterations in hippocampal development. For example, a series of studies has demonstrated that maternal support in early childhood predicts greater hippocampal volume and more rapid hippocampal development in mid to late childhood (Luby et al., 2012, 2016). Further, Blankenship et al. (2019) found that positive parenting of 4-year-olds predicted greater hippocampal head volumes 3 years later. There are, however, some null findings and findings in the opposite direction surrounding caregiving and the hippocampus. Some studies suggest positive caregiving experiences relate to *smaller* hippocampal volumes in infants (Rifkin-Graboi et al., 2015) and toddlers (Bernier et al., 2019), and other studies reveal no relation between maternal caregiving and hippocampal volume in early (Lee et al., 2019) and middle childhood (Kok et al., 2015). Inconsistent findings, combined with the crucial role of the hippocampus in children’s cognitive and emotional well-being (e.g., Barch et al., 2019), highlight the need to continue examining relations between caregiving and the hippocampus.

The amygdala is another structure involved in stress-regulation (Callaghan and Tottenham, 2016a). Specifically, research suggests that the amygdala is one of several forebrain regions that plays a critical role in upregulating the HPA axis following the onset of a stressful event (Honkaniemi et al., 1992). Research suggests that early exposure to negative environments is associated with larger amygdala volume (Tottenham et al., 2010; Roth et al., 2018)—a structural difference that has been attributed to accelerated amygdala maturation in threatening contexts (Tottenham et al., 2010). Although it is important to note that the vast majority of this research focuses on neglected and maltreated samples (Belsky and de Haan, 2011), some research corroborates these findings in typically developing community samples without histories of maltreatment or neglect. In one study, maternal sensitivity marginally related to smaller right and left amygdala volumes in infants (Rifkin-Graboi et al., 2015). Further, research found that mothers’ greater punishing responses predicted young adolescents’ larger amygdala volumes (Whittle et al., 2009). Moreover, one study demonstrated that maternal depression, often associated with deficits in caregiving (Lovejoy et al., 2000), was associated with larger amygdala volumes in late childhood (Lupien et al., 2011). Longitudinal research has demonstrated that maternal sensitivity in toddlers predicted smaller right amygdala volumes in late childhood (Bernier et al., 2019), maternal sensitivity in infancy predicted boys’ smaller amygdala volumes at age six (Lee et al., 2019), and positive parenting predicted less amygdala growth from early to mid-adolescence (Whittle et al., 2014). Some longitudinal work, however, has failed to demonstrate relations between parental sensitivity in early childhood and amygdala volume in middle childhood (Kok et al., 2015). Thus, further research is necessary to reconcile conflicting findings and strengthen confidence in the relations between normative variations in caregiving and children’s amygdala volume.

In addition to findings in subcortical structures, some research indicates that normative variations in maternal caregiving relate to variations in the cortex. It is important to note, however, that much of this limited literature involves adolescents and young adults. For example, maternal aggression predicted greater thickening of the superior frontal gyrus and the right parietal lobe in males from early adolescence to early adulthood (Whittle et al., 2016), and mothers’ punishing responses predicted greater dorsal anterior cingulate cortex and orbito-frontal cortex volumes in young adolescents (Whittle et al., 2009). Further, young adults’ self-reports of maternal warmth predicted greater gray matter volume (GMV) in the prefrontal cortex (Yang et al., 2018). Null findings exist as well. In one study, researchers failed to find relations between parental nurturance in early to middle childhood and cortical thickness in any brain structures in late adolescence (Avants et al., 2015).

Although limited, some research has demonstrated relations between infants’ and children’s cortical brain structure and normative caregiving. For example, evidence suggests that mothers’ negative affect relates to infants’ smaller total gray and white matter volumes (Sethna et al., 2017), and parental praise in middle childhood relates to greater GMV in the insula (Matsudaira et al., 2016). In addition, parental sensitivity in early childhood predicted greater brain volume and total GMV in middle childhood (Kok et al., 2015). An intervention study, however, failed to find any effects of a parent sensitivity program on infants’ total and regional GMV s (Milgrom et al., 2010). Promising early research indicates that some relations between caregiving and children’s cortical structures exist. Yet findings about *which* cortical structures relate to caregiving and the exact *nature* of these relations have not yet converged, possibly due to the limited number of studies. Thus, the present study aimed to help the field better understand how parenting becomes biologically embedded in the developing brain.

The present study emerged from the following: (a) compelling evidence that mothers’ attachment representations relate to their caregiving behaviors, (b) sparse and somewhat inconsistent evidence that normative variation in mothers’ caregiving behaviors relates to individual differences in children’s brain structure, and (c) the lack of direct examination of links between mothers’ attachment representations and children’s brain structure. As such, the present study had two principal goals: (1) To examine relations between mothers’ attachment representations and children’s brain structure, and (2) to examine the indirect effect of mothers’ attachment representations on children’s brain structures through one aspect of mothers’ caregiving, responses to children’s distress. We included data from 52 children (ranging in age from 3 to 8 years) and their mothers. Mothers completed the Attachment Script Assessment (ASA; Waters and Rodrigues-Doolabh, 2001) and the Experiences in Close Relationships Scale (ECR; Brennan et al., 1998) to assess attachment representations. Mothers also completed the Coping with Children’s/Toddlers’ Negative Emotions Scales to assess responses to distress and children completed an magnetic resonance imaging (MRI) scan.

Our pre-registered hypotheses^1^ were that: (1) mothers’ secure base script knowledge would relate positively to their children’s hippocampal volumes and negatively to their children’s amygdala volumes, (2) mothers’ attachment avoidance and attachment anxiety would relate negatively to their children’s hippocampal volumes and positively to their children’s amygdala volumes, and (3) mothers’ unsupportive responses to children’s distress would facilitate indirect links between mothers’ attachment representations (secure base script knowledge, attachment avoidance, and attachment anxiety) and amygdala and hippocampal volumes. Our pre-registered exploratory analyses examined whether: (1) mothers’ attachment representations would relate to global metrics of brain development, including children’s intracranial volume, total gray and white matter volume, subcortical GMV, and patterns of regional cortical thickness and surface area, and (2) mothers’ supportive responses toward children’s distress would facilitate indirect relations between mothers’ attachment representations and children’s brain structure. We had no *a priori* hypotheses about whole brain metrics or regional cortical thickness and surface area due to the paucity of research and inconsistent findings on early normative caregiving experiences and children’s cortical structure. We focused our confirmatory analyses on unsupportive, as opposed to supportive, responses toward distress. This was due to research demonstrating the relatively greater link between unsupportive responses and mothers’ insecure attachment (Jones et al., 2014), and between unsupportive responses and children’s poor social-emotional functioning (e.g., Eisenberg et al., 1999; Fabes et al., 2001; Perry et al., 2012; Shewark and Blandon, 2015), both aspects of functioning that relate to differences in brain structure (Gee, 2016). However, supportive responses were also examined in exploratory analyses.

## Materials and Methods

Participants were drawn from two larger longitudinal studies investigating memory and brain development (see Botdorf and Riggins, 2018; Allard et al., 2019; Canada et al., 2019). Dyads were included in the present study if the mother had completed the Attachment Script Assessment (Waters and Rodrigues-Doolabh, 2001).

### Study 1

#### Participants

Participants were 36 mothers (*M*_*age*_ = 33.23, *SD* = 5.03) and their 4- to 8-year-old children (20 males; *M*_*age*_ = 6.66, *SD* = 1.29) taken from a larger longitudinal study examining behavioral and brain development during early childhood. The majority of children (77%) identified as White, 11.5% identified as African American, and 11.5% identified as multiracial. Regarding ethnicity, 9.1% were Latinx/Hispanic. The majority of families were affluent: 79% of families had an average household income of more than $95,000/year; 86.1% of participants had a parent with at least a college degree, and 50% of participants had a parent with a post-graduate degree (see Table 1 for more demographic information). Participants were recruited from the Baltimore-Washington metropolitan area through flyers, online advertisements, and a University-maintained database of families interested in participating in research.

**TABLE 1:** Demographics of participants in Study 1 and Study 2.

	Study 1	Study 2	Total
	(*n* = 36)	(*n* = 16)	(*n* = 52)
	*n* (%) or *M* (*SD*)	*n* (%) or *M* (SD)	*n* (%) or *M* (SD)
**Mothers’ age**	33.23 (5.03)	38 (5.21)	34.29 (5.40)
**Children’s age**	6.66 (1.29)	4.81 (0.49)	6.12 (1.40)
**Children’s sex**			
Male	20 (55.6%)	8 (50%)	28 (53.8%)
Female	16 (44.4%)	8 (50%)	24 (46.2%)
**Children’s ethnicity**			
Hispanic	3 (8.3%)	3 (18.8%)	6 (11.5%)
Non-hispanic	32 (88.9%)	8 (50.0%)	40 (76.9%)
Not reported	1 (2.8%)	5 (31.2%)	6 (11.5%)
**Children’s race**			
White	27 (75%)	11 (68.8%)	38 (73.1%)
African American	4 (11.1%)	0 (0%)	4 (7.7%)
Multiracial	4 (11.1%)	0 (0%)	4 (7.7%)
Not reported	1 (2.8%)	5 (31.2%)	6 (11.5)
**Mothers’ ethnicity**			
Hispanic	3 (88.9%)	3 (18.8%)	6 (11.5%)
Non-hispanic	32 (8.3%)	8 (50.0%)	40 (76.9%)
Not reported	1 (2.8%)	5 (31.2%)	6 (11.5%)
**Mothers’ education**			
High School	0 (0%)	0 (0%)	0 (0%)
Some College	1 (2.8%)	0 (0%)	1 (1.9%)
Technical or Associates Degree	4 (11.1%)	0 (0%)	4 (7.7%)
College	10 (27.8%)	1 (6.2%)	11 (21.2%)
Some Graduate School	3 (8.3%)	0 (0%)	3 (5.8%)
Post-Graduate Degree	18 (50%)	10 (62.5%)	28 (53.8%)
Not reported	0 (0%)	5 (31.2%)	5 (9.6%)

Exclusionary criteria were: children’s history of head trauma or brain abnormality, abnormal circadian function, neurological disorders, premature birth, diagnosis of ADHD or other learning disability, diagnosis of psychiatric disorders, history of developmental delays or disorders, family history of autism spectrum disorder, child or parent was not English-speaking, and contraindications for MRI (e.g., metal in the body).

#### Procedures

Mothers were provided with a personal link to complete the online questionnaires about their attachment style (anxiety and avoidance), their responses to children’s distress, and demographic information. Mothers were allowed to complete this survey at home or in the lab while their child was participating in the study. Participants visited the university for two visits, typically within 1 week of each other. During the first visit, children participated in an MRI scan. During the second visit, mothers completed the Attachment Script Assessment (ASA; Waters and Rodrigues-Doolabh, 2001; Waters and Waters, 2006) to assess secure base script knowledge while their children participated in memory tasks unrelated to the present study. Mothers were compensated with cash and children were given a small prize for their participation.

#### Measures

##### Magnetic Resonance Imaging Acquisition

To prepare for MR data acquisition, participants were familiarized with the scanner environment using a mock MRI scanner. Children were provided motion feedback and practiced lying still during the mock scan. Following practice in the mock scanner, children were scanned using a Siemens 3.0T scanner (MAGNETOM Trio Tim System, Siemens Medical Solutions, Erlangen, Germany) with a 32-channel coil. Given the high possibility of movement in younger populations, additional padding was placed around each participant’s head to reduce head movement. Children also watched a movie during the scan to enhance compliance. A high resolution (0.9 mm^3^) T1-weighted whole brain structural scan consisting of 176 contiguous sagittal slices (1900 ms TR; 2.32 ms TE; 900 ms inversion time; 9° flip angle; pixel matrix = 256 × 256) was acquired during imaging. To ensure high image quality, images were visually inspected following the scan. If the image quality was deemed low, the scan was repeated.

##### Magnetic Resonance Imaging Analysis

Preprocessing of structural T1-weighted images consisted of image registration, skull stripping, smoothing, motion correction, and subcortical segmentation via Freesurfer (v5.1^2^). Hippocampal and amygdala volumes were obtained and adjusted via the automated segmentation adapter tool^3^ (Wang et al., 2011) and split into subregions using standard anatomical landmarks (Riggins et al., 2015). To obtain quality measures of cortical thickness and surface area, two trained coders checked the boundary lines separating white and pial surfaces. In the case of errors, such as slices where the gray/white matter boundary extended past the skull or the pial boundary encapsulated portions of the skull, editors corrected these boundaries. Corrections were only made if the error persisted for more than 7 contiguous slices (Botdorf and Riggins, 2018). Alterations were first made by changing the watershed value in FreeSurfer. If this step did not eliminate the error, then manual edits were made (Ducharme et al., 2014). Edits were made for approximately 58% of the sample and involved an average of 14.6 slices (range 9–100). After all corrections were made, cortical thickness and surface area were calculated using FreeSurfer (Fischl and Dale, 2000). Freesurfer was also used to extract total and subcortical GMV (Fischl et al., 2002).

##### Attachment Script Assessment

Mothers were instructed to create a story, using a 12-to-14-word list as a guide (Waters and Rodrigues-Doolabh, 2001; Waters and Waters, 2006). The procedure includes two stories about parent–child relationships (e.g., a parent and child at the doctor’s office) and two stories about adult–adult relationships (e.g., a couple on a camping trip).

Stories were coded on a 7-point scale indicating the extent to which the story demonstrates secure base script knowledge, from 7 (*extensive secure base script knowledge*) to 1 (*absence of secure base script knowledge*). High scores reflect stories wherein an individual becomes distressed, seeks and receives care from a supportive caregiver (in the parent–child stories) or a supportive partner (in the adult–adult stories), is comforted as a function of that care, and is able to resume activities. The four scores from each participant were averaged to create a mean score of secure base script knowledge. All stories were coded by two masked coders who were trained to reliability by a developer of the task (Harriet Waters) and demonstrated strong interrater reliability (α = 0.88).

##### Experiences in Close Relationships Scale

Mothers completed the general form of the widely used 36-item self-report measure of adult attachment anxiety and avoidance; this version assesses these dimensions with respect to close relationships generally, rather than asking specifically about romantic relationships (Brennan et al., 1998). The avoidance dimension reflects individuals’ feelings of discomfort with close relationships and avoidance of intimacy or reliance on others (e.g., “I get uncomfortable when someone wants to be very close to me”), whereas the anxiety dimension reflects individuals’ fear of interpersonal rejection and abandonment (e.g., “I worry about being rejected or abandoned”). Each item is rated on a 7-point scale from 1 (*strongly disagree*) to 7 (*strongly agree*), and some items are reverse-scored so that higher scores represent greater avoidance or anxiety. Mothers’ attachment anxiety and avoidance were calculated by averaging responses across subscale items. Good internal consistency was evident (anxiety ω = 0.93, avoidance, ω = 0.94).

##### Coping With Children’s Negative Emotions Scale

This questionnaire was used to measure mothers’ unsupportive responses to child distress. Participants rated their likelihood of engaging in each of 6 possible responses to their children’s negative emotions in 12 hypothetical scenarios about a child in distress (e.g., “If my child falls off his bike and breaks it, and then gets upset and cries, I would…”) (Fabes et al., 1990). For each scenario, caregivers rated each possible response from 1 (*very unlikely*) to 7 (*very likely*). Following Fabes et al. (2002), we created two indices: supportive and unsupportive responses to distress.

For each scenario, unsupportive responses include the following: (1) distress reactions (e.g., “Remain calm and not let myself get anxious” [reverse-scored]); (2) punitive reactions (e.g., “Tell my child that if he doesn’t stop crying, he won’t be allowed to ride his bike anytime soon”); and (3) minimizing reactions (e.g., “Tell my child that he is over-reacting”).

For each scenario, supportive responses include the following: (1) expressive encouragement (e.g., “Tell my child it’s OK to cry”); (2) emotion-focused reactions (e.g., “Comfort my child and try to get him to forget about the accident”); and (3) problem-focused reactions (e.g., “Help my child figure out how to get the bike fixed”). The subscales demonstrated strong reliability and construct validity in previous research (Fabes et al., 2002; Shewark and Blandon, 2015) and strong reliability in the present sample (unsupportive ω = 0.92, supportive ω = 0.95). Reliability reported for the unsupportive subscale does not include one item from the punitive subscale (“Send my child to his or her room to cool off”) because a reliability statistic could not be computed due to missingness. Subscale reliability without this item is very strong (ω = 0.92); as we did throughout all analyses, we utilized participant mean imputation for missing values for this item.

### Study 2

#### Participants

Participants were 16 mothers (*M_*age*_* = 38, *SD* = 5.21) and their 3- to 5-year-old children (8 males; *M*_*age*_ = 4.81, *SD* = 0.49) taken from a larger longitudinal study examining behavioral and brain development during early childhood. All parents identified their children as White. Regarding ethnicity, 27% were Latinx/Hispanic. The majority of families were affluent. All had an average household income of more than $95,000/year, all had a parent with at least a college degree, and 90.1% had a parent with a post-graduate degree (see Table 1 for additional demographic information). Recruitment procedures and inclusion and exclusion criteria were the same as in Study 1.

#### Procedure

The measures and procedures in Study 2 were identical to those in Study 1 with the following exceptions: (a) Study 2 participants completed the Coping with Toddlers’ Negative Emotions Scale (CTNES; Fabes et al., 1990) due to differences in children’s ages between Studies 1 and 2; (b) Study 2 participants completed the short form of the Experiences in Close Relationships scale (ECR-S, Wei et al., 2007); (c) Study 2 research sessions typically took place within 2 weeks of one another; (d) Study 2 mothers always completed the ASA at home, never in the lab because the child participated in the study from the home; (e) Study 2 participants received additional instructions and practice to prepare them for the scan due to their younger ages; (f) Study 2 MRI Images were analyzed using Freesurfer version 6.0.0 (see footnote 2; Fischl et al., 2002; Fischl, 2012).

#### Measures

##### Magnetic Resonance Imaging Acquisition

In Study 2, participants completed the same MR acclimation task and data acquisition procedures as in Study 1 with one additional step. Prior to their visit, Study 2 children completed an additional at-home MR acclimation task to increase scan success rate: Children lay in a fabric tunnel while they listened to MR noises and motion feedback from an experimenter.

##### Magnetic Resonance Imaging Analysis

Hippocampal and amygdala volumes were obtained via Freesurfer 6.0 (see footnote 2) and adjusted via the automated segmentation adapter tool (see footnote 3; Wang et al., 2011). Previous research has suggested that although different versions of Freesurfer produce nominally different values for subcortical volumes and cortical thickness (e.g., Gronenschild et al., 2012), ultimately these variations do not change outcomes in correlational research (Chepkoech et al., 2016; Bigler et al., 2020). We explored the potential effects of using different versions of Freesurfer in Study 1 and 2 by re-processing the cases from Study 2 in version 5.1. We then ran correlational analyses between volumes obtained from version 5.1 and 6.0 (*r*s ranged from 0.84 to 0.99) and replaced values obtained from version 6.0 with values from 5.1 in analyses that yielded significant effects (i.e., thalamus and amygdala). All results were similar, supporting our conclusion that the variation in Freesurfer version was not driving the observed effects. We retained the values from 5.1 as they were subjected to a more rigorous quality control procedure that was similar across the two studies.

##### Experiences in Close Relationships Scale—Short Form

Mothers completed a self-report measure of adult attachment anxiety and avoidance in close relationships (six anxiety items, five avoidance items; mean scores calculated for each subscale) (Wei et al., 2007). Each item is rated on a 7-point scale from 1 (*strongly disagree*) to 7 (*strongly agree*). ECR-S yields reliable and valid scores, with high correlations between the short and original versions for both anxiety (*r* = 0.94) and avoidance *(r* = 0.95) subscales (Wei et al., 2007). Although the ECR-S is a 12-item measure, one avoidance item was accidentally omitted (“I find that my partners don’t want to get as close as I would like”); however, the measure still demonstrated good internal consistency in the present study (anxiety, ω = 0.78, avoidance, ω = 0.90).

##### Coping With Toddlers’ Negative Emotions Scale

This questionnaire mirrors the Coping with Children’s Negative Emotions Scale (CCNES) and is adapted to reflect scenarios with toddlers (Spinrad et al., 2004). Following Gudmundson and Leerkes (2012), we created two indices: supportive and unsupportive responses to distress. Studies support the reliability and construct validity of the CTNES in preschool children (e.g., Eisenberg et al., 2010; Gudmundson and Leerkes, 2012), and both subscales demonstrated strong reliability in the present study (unsupportive ω = 0.93, supportive ω = 0.96).

#### Statistical Analyses

Our full data analysis plan was pre-registered (see footnote 1). Consistent with this plan, we first examined race (coded as White and non-White) and household income as possible empirically derived covariates. We used Kendall’s Tau to explore whether household income related to amygdala or hippocampal volumes. We used independent samples *t*-tests to determine whether race (dichotomized as White or non-White) related to amygdala, and hippocampal volumes. None of the bivariate relations was significant (all *ps* > 0.05); thus, race and household income were not utilized as covariates. We included the *a priori* covariates of child age and sex in all of the following analyses because we expected them to relate to amygdala and hippocampal volumes; as predicted, both age and sex were significantly related to the brain measures of interest (see Table 2). Next, we used Pearson’s correlations to examine whether any of our three attachment representation variables (anxiety, avoidance, and secure base script knowledge) related to intracranial volume (referred to as estimated Total Intracranial Volume or eTIV), total GMV, subcortical GMV, or white matter volume (WMV). None of the bivariate correlations was significant (all *ps* > 0.05); thus eTIV, total gray matter, subcortical gray matter, and white matter volume were not utilized as covariates in the primary analyses.

**TABLE 2:** Bivariate correlations among main study variables.

	Variable	1	2	3	4	5	6	7	8
**Child demographics**									
	Sex (Male)^a^								
	Age	0.40**							
**Child brain structure**									
	Hipp	0.46**	0.58**						
	Amy	0.67**	0.61**	0.68**					
	L Amy	0.55**	0.63**	0.69**	0.91**				
	R Amy	0.66**	0.49**	0.56**	0.93**	0.70**			
	Thal	0.45**	0.31*	0.48**	0.55**	0.52**	0.49**		
	Lat Occ	0.33	0.08	0.11	0.27	0.27	0.24	0.23	
	eTIV	0.69**	0.36*	0.48**	0.68**	0.60**	0.65**	0.63**	0.53**
	WMV	0.63**	0.57**	0.57**	0.81**	0.75**	0.73**	0.67**	0.42**
	GMV	0.49**	0.45**	0.51**	0.68**	0.61**	0.65**	0.59**	0.56**
	SGMV	0.09	0.67**	0.64**	0.62**	0.65**	0.50**	0.31*	0.03
**Maternal attachment representations and parenting**									
	SBS	0.12	0.01	–0.05	–0.15	–0.18	–0.09	−0.34*	–0.05
	Anx	0.09	–0.10	–0.12	–0.17	–0.11	–0.20	−0.31*	0.04
	Avo	0.08	–0.07	0.00	–0.08	–0.02	–0.12	–0.18	0.04
	Unsup	0.04	–0.06	–0.09	0.04	0.02	0.06	0.07	0.26
	Sup	–0.01	–0.05	–0.12	–0.11	–0.09	–0.11	0.00	–0.16

	**Variable**	**9**	**10**	**11**	**12**	**13**	**14**	**15**	**16**

**Child demographics**									
	Sex (Male)^a^								
	Age								
**Child brain structure**									
	Hipp								
	Amy								
	L Amy								
	R Amy								
	Thal								
	Lat Occ								
	eTIV								
	WMV	0.88**							
	GMV	0.88**	0.82**						
	SGMV	0.42**	0.62**	0.56**					
**Maternal attachment representations and parenting**									
	SBS	–0.16	–0.23	–0.16	–0.10				
	Anx	–0.07	–0.09	–0.17	–0.05	0.08			
	Avo	–0.13	–0.10	–0.06	–0.06	0.03	0.50**		
	Unsup	0.15	0.08	0.17	–0.02	–0.10	0.24	0.20	
	Sup	–0.04	–0.04	–0.14	–0.14	–0.10	−0.45**	–0.24	−0.40**

*Our a priori regions of interest were the hippocampus and the amygdala.*

*^a^All correlations with child sex (coded as 1 = Female, 2 = Male) are biserial.*

*Hipp, Hippocampal Volume; Amy, Total Amygdala Volume; L Amy, Left Amygdala Volume; R Amy, Right Amygdala Volume; Thal, Thalamus Volume; Lat Occ, Lateral Occipital Cortex Volume; eTIV, Intracranial Volume; WMV, White Matter Volume; GMV, Gray Matter Volume; SGMV, Subcortical Gray Matter Volume; SBS, Secure Base Script Knowledge; Anx, Attachment Anxiety; Avo, Attachment Avoidance; Unsup, Unsupportive Responses to Distress; Sup, Supportive Responses to Distress.*

*p < 0.05; **p < 0.01.

Second, we conducted multiple regression analyses using Mplus version 5.2 (Muthén and Muthén, 1998-2007) to test our pre-registered hypotheses that mothers’ attachment representations would relate to bilateral amygdala and hippocampal volumes, and to test our follow-up exploratory questions about relations among attachment representations and unilateral amygdala volumes and hippocampal subregions. Following Mueller and Hancock (2010), we utilized full information maximum likelihood estimation (FIML) to handle missing data. We used participant mean imputation to estimate scores for participants with missing item responses on a given scale, a statistically sound technique when less than 10% of item scores are missing (Schafer and Graham, 2002; Parent, 2013). To control for the potential of type 1 error, we conducted sensitivity analyses utilizing certain regions of the brain we did not expect to relate to caregiving/attachment representations (the thalamus and occipital cortex, see Humphreys et al., 2018).

Third, we conducted analyses of indirect effects using Mplus’ MODEL INDIRECT procedure (Stride et al., 2015). This procedure utilizes bootstrapping methods to estimate confidence intervals around the indirect effect. As recommended by Preacher and Hayes (2008) analyses were conducted with 5,000 bootstrapped samples. We used this procedure to test our pre-registered hypotheses regarding indirect effects of mothers’ attachment representations on children’s bilateral hippocampal and amygdala volumes through unsupportive responses to children’s distress, and to test our follow-up exploratory questions about the above indirect effects through supportive responses.

Finally, we conducted an exploratory vertex-wise whole-brain analysis in Freesurfer’s Qdec application to test links between mothers’ attachment representations, cortical thickness, and surface area. Monte Carlo simulations were used to correct for multiple comparisons. The final whole-brain significance threshold was set at *p* < 0.05.

## Results

### Descriptive Statistics and Missing Data

Table 2 displays bivariate correlations among all major study variables and Table 3 presents descriptive statistics for all major study variables. Out of the 52 participants, three were missing MRI data, seven were missing attachment avoidance and anxiety, and six were missing supportive and unsupportive responses to children’s distress. Missing values were handled with FIML.

**TABLE 3:** Descriptive statistics of main study variables.

Variable	*N* (%)	*M*	*SD*	Range	Skewness
**Demographics**					
Sex (Male)	28 (53.85)				
Age (years)		6.12	1.4	4.03–8.93	0.42
**Brain structure (mm^3^)**					
Hipp		6517.9	665.12	5225.0–8078.0	0.43
Amy		3105.88	363.76	2177.7–4017.0	0.20
L Amy		1478.41	186.94	1008.0–1839.0	–0.22
R Amy		1627.46	208.06	1169.7–2197.0	0.44
Thal		14085.87	1318.48	11516.0–16810.0	0.25
Lat Occ		29986.35	3411.55	23187.0–38142.0	0.47
eTIV		1379660.6	120800.4	1165227.0–1645938.0	0.49
WMV		415242.98	57169.16	310838.00–549534.20	0.37
GMV		787785.67	63188.96	658818.5–951868.2	0.34
SGMV		152868.27	65318.97	44747.0–234247.0	–0.78
**Attachment and parenting**					
SBS		3.51	0.98	1.00–6.33	
Anx		2.7	1.04	1.22–5.11	0.29
Avo		2.34	1.00	1.00–4.50	0.31
Unsup		2.34	0.62	1.31–3.90	0.47
Sup		5.47	0.85	2.11–6.78	–1.35

*Hipp, Hippocampal Volume; Amy, Total Amygdala Volume; L Amy, Left Amygdala Volume; R Amy, Right Amygdala Volume; Thal, Thalamus Volume; Lat Occ, Lateral Occipital Cortex Volume; eTIV, Intracranial Volume; WMV, White Matter Volume; GMV, Gray Matter Volume; SGMV, Subcortical Gray Matter Volume; SBS, Secure Base Script Knowledge; Anx, Attachment Anxiety; Avo, Attachment Avoidance; Unsup, Unsupportive Responses to Distress; Sup, Supportive Responses to Distress.*

### Mothers’ Attachment Representations and Children’s Amygdala and Hippocampal Volumes

**FIGURE 1:**
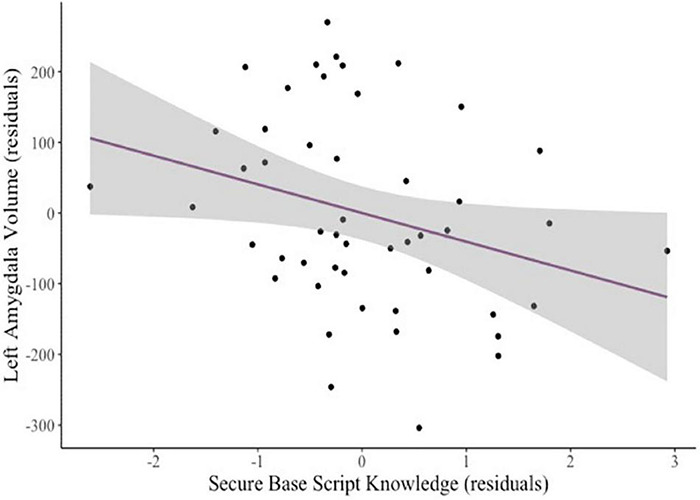
Added variable plot displaying the partial correlation between mothers’ secure base script knowledge and children’s left amygdala volume controlling for covariates (sex and age). The shaded region reflects the 95% confidence interval around the regression line.

After controlling for child age and sex, mothers’ secure base script knowledge marginally predicted smaller bilateral amygdala volumes (β = –0.19, *p* = 0.060). To explore this finding further, we examined unilateral amygdala volumes. Greater secure base script knowledge predicted smaller left (β = –0.21, *p* = 0.031; see Figure 1) but not right (β = –0.13, *p* = 0.229) amygdala volumes. Neither attachment anxiety (β = –0.14, *p* = 0.252) nor attachment avoidance (β = –0.05, *p* = 0.718) predicted bilateral amygdala volumes. There were no significant relations between secure base script knowledge, anxiety, or avoidance and bilateral hippocampal volumes (all *ps* > 0.295). To explore possible associations between mothers’ attachment representations and hippocampal subregions, we examined whether secure base script knowledge, anxiety, and avoidance related to hippocampal head, body, and tail volumes. There were no significant relations between any measures of attachment representations and hippocampal head, body, or tail volumes; however, secure base scripts knowledge marginally predicted smaller hippocampal head volumes (β = –0.21, *p* = 0.078), but anxiety (β = –0.18, *p* = 0.243) and avoidance (β = 0.03, *p* = 0.876) did not.

**FIGURE 2:**
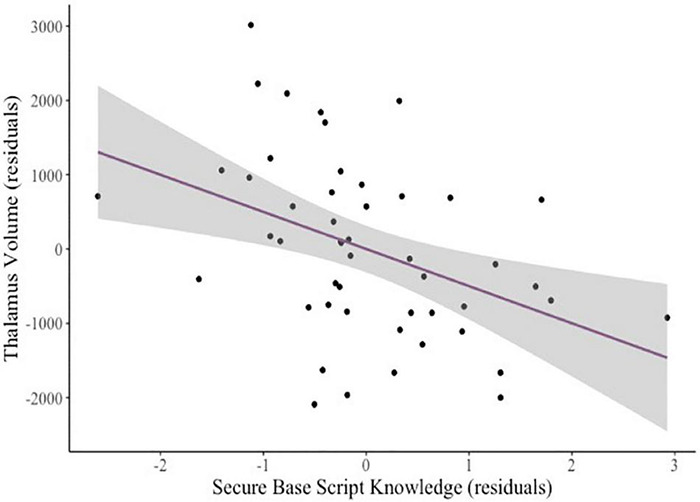
Added variable plot displaying the partial correlation between mothers’ secure base script knowledge and children’s thalamus volume controlling for covariates (sex and age). The shaded region reflects the 95% confidence interval around the regression line.

In a pre-registered follow up sensitivity analysis to explore specificity of the associations of secure base script knowledge and amygdala volume, we examined two regions we did not expect to relate to secure base script knowledge: The occipital cortex and the thalamus (Humphreys et al., 2018). As expected, there was no relation between secure base script knowledge and occipital cortex volume (β = –0.08, *p* = 0.548). However, contrary to our expectations, greater secure base script knowledge (β = –0.37, *p* = 0.001; see Figure 2) and greater attachment anxiety (β = –0.30, *p* = 0.039) predicted smaller thalamus volume.

### Indirect Effects of Mothers’ Attachment Representations on Children’s Brain Structure Through Responses to Distress

We tested whether mothers’ self-reported caregiving in response to children’s distress explained indirect relations between attachment representations and bilateral hippocampal and amygdala volumes. There were no significant indirect effects of secure base script knowledge, attachment avoidance, or attachment anxiety on bilateral amygdala volumes or hippocampal volumes through unsupportive responses to distress (all *ps* > 0.649). The same pattern held when exploring the role of supportive responses to children’s distress (all *ps* > 0.232). There were also no significant direct links between bilateral hippocampal and amygdala volume and either supportive or unsupportive responses to distress (all *p*s > 0.402). Mothers’ attachment anxiety predicted less supportive responses to distress (β = –0.45, *p* = 0.001). Contrary to our predictions, neither secure base script knowledge nor attachment avoidance predicted supportive or unsupportive responses to distress (all *ps* > 0.271).

### Mothers’ Attachment Representations and Children’s Whole Brain Measures

**FIGURE 3:**
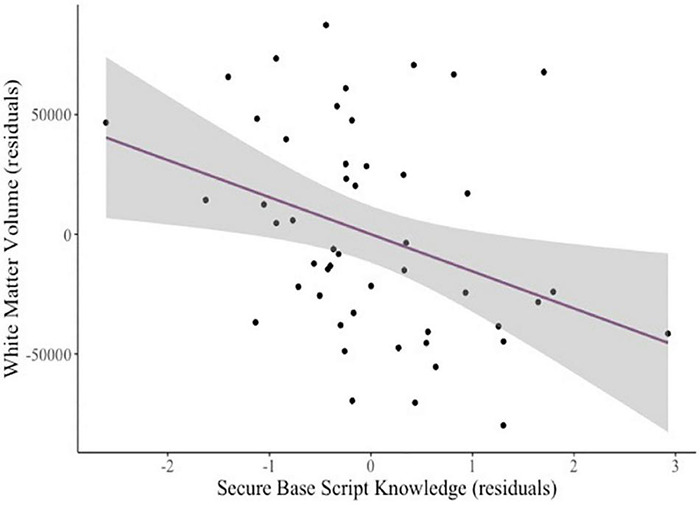
Added variable plot displaying the partial correlation between mothers’ secure base script knowledge and children’s white matter volume controlling for covariates (sex and age). The shaded region reflects the 95% confidence interval around the regression line.

To explore whether mothers’ attachment representations related to whole brain metrics, we tested relations among our attachment predictors and intracranial volume (eTIV), total GMV, subcortical GMV, and WMV controlling for age and sex. There were no significant relations between eTIV and secure base script knowledge (β = –0.19, *p* = 0.108), attachment anxiety (β = –0.02, *p* = 0.904), or attachment avoidance (β = –0.16, *p* = 0.264). There were also no significant relations between total GMV and secure base script knowledge (β = –0.19, *p* = 0.106), attachment anxiety (β = –0.16, *p* = 0.266) or attachment avoidance (β = 0, *p* = 1.00). No significant relations between attachment predictors and subcortical GMV emerged (all *ps* > 0.409). There was a significant negative relation between WMV and secure base script knowledge (β = –0.27, *p* < 0.009; see Figure 3) but not attachment anxiety (β = –0.03, *p* = 0.788) or avoidance (β = –0.12, *p* = 0.384).

To explore whether a whole brain effect may be contributing to the relation between secure base script knowledge and left amygdala volume and thalamus volume, we added eTIV to the models predicting left amygdala volume and thalamus volume, controlling for child sex and age. When including eTIV in the model, the relation between secure base script knowledge and the left amygdala became non-significant (β = –0.15, *p* = 0.108). The relation between secure base script knowledge and thalamus volume, however, remained significant when controlling for eTIV (β = –0.29, *p* = 0.006).

Finally, consistent with our pre-registered approach, we utilized a vertex-by-vertex analysis to determine whether mothers’ attachment representations related to children’s cortical thickness and surface area. The vertex-by-vertex whole brain analysis did not demonstrate any relations among our attachment predictors of interest and cortical thickness. However, greater secure base script knowledge predicted greater surface area of the right pericalcarine cortex (*p* < 0.05). This pattern of results remained the same when the 14 subjects who had the largest numbers of edits (i.e., greater than the mean or more than 15) were removed.

## Discussion

**FIGURE 4:**
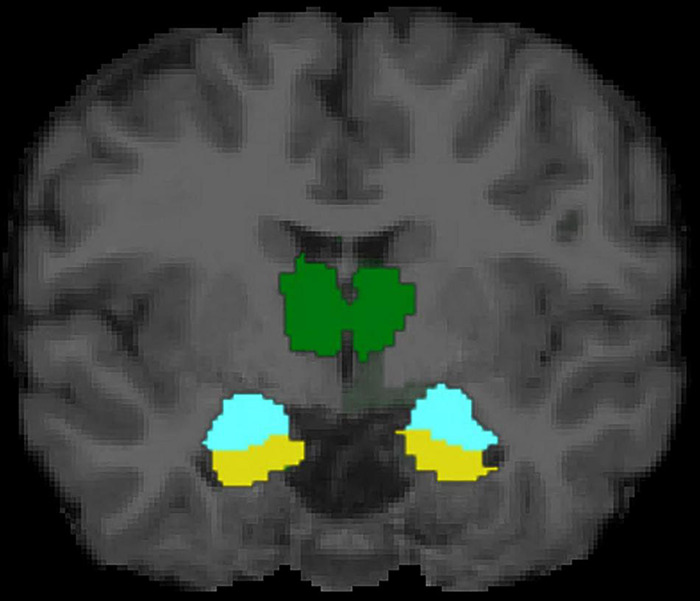
Subcortical structures of interest in mid-coronal view. Hippocampus depicted in yellow, amygdala depicted in turquoise, and thalamus depicted in green.

**FIGURE 5:**
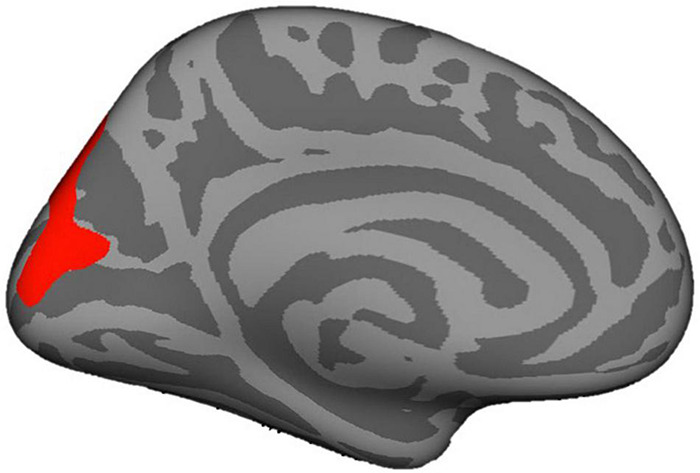
Pericalcarine cortex depicted in red.

The present study is first to our knowledge to examine links between mothers’ attachment representations and children’s brain structure, and to explore potential indirect effects via caregiving behavior in response to child distress. In a non-clinical sample, we examined two conceptualizations of mothers’ attachment representations: self-reported attachment style and secure base script knowledge (i.e., mothers’ procedural knowledge of how a secure base is provided in times of need; Waters and Waters, 2006). Mothers’ greater secure base script knowledge, but not attachment style, was associated with smaller left amygdala volume in early childhood. In contrast, mothers’ attachment representations were not significantly related to children’s hippocampal volume. Pre-registered exploratory analyses revealed that mothers’ greater secure base script knowledge was also associated with children’s smaller WMV, smaller thalamus volume, and larger surface area of the right pericalcarine cortex, whereas maternal representations were unrelated to cortical thickness (see Figures 4, 5 for visualizations of cortical and subcortical structures). Contrary to predictions, we observed no indirect effects via caregiving behavior in response to child distress. Findings highlight the role that parental attachment representations—particularly secure base script knowledge—may play in children’s brain structure and add to a growing literature on the ways in which parental factors shape the developing brain in childhood (Belsky and de Haan, 2011; Farber et al., 2020). We discuss each finding in turn and outline directions for future research.

The finding that mothers’ secure base script knowledge was associated with children’s smaller left amygdala volume (and marginally to smaller bilateral volume) provides partial support for our hypotheses. Mothers’ secure base script knowledge involves a schema for the effective co-regulation of distress and protection from potential threats in the child’s environment (Waters and Waters, 2006). Children’s experiences of sensitive care to help manage distress, particularly in times of threat, help them learn to self-regulate (Hofer, 1995; Luecken and Lemery, 2004; Köhler-Dauner et al., 2019). Indeed, the function of a secure base is to protect children from threat (Bowlby, 1969/1982), and experiences of secure base provision (guided by mothers’ scripts) may be especially important for the development of neurobiological systems involved in threat responding, including the amygdala (Cassidy et al., 2013). In addition, mothers’ secure base script knowledge has been linked to child secure attachment (e.g., Bost et al., 2006), and secure attachment is a robust predictor of self-regulation (see Cassidy, 1994; Calkins and Leerkes, 2011). Thus, maternal secure base script knowledge may be particularly important for the development of neural circuits involved in stress reactivity and self-regulation. This aligns with the theory that the extent to which sensitive caregivers can co-regulate children’s distress shapes amygdala development (Callaghan and Tottenham, 2016a) and children’s physiological responses to stressful situations (Köhler-Dauner et al., 2019), and with research linking negative parenting to accelerated maturation and larger amygdala volume on the one hand (Tottenham et al., 2010) and positive parenting to smaller amygdala volume on the other hand (Whittle et al., 2014; Rifkin-Graboi et al., 2015). Together, findings suggest that the full spectrum of parenting experiences—not only adversity but also normative variation in parental characteristics—merit attention to understand ways in which the social environment is reflected in brain structure (Belsky and de Haan, 2011; Farber et al., 2020).

Although we had *a priori* hypotheses regarding links between amygdala volume and mothers’ secure base script knowledge driven by theory (Cassidy, 1994; George and Solomon, 2008; Callaghan and Tottenham, 2016a) and empirical research (Tottenham et al., 2010; Rifkin-Graboi et al., 2015), it is important to note that our hypotheses involved *bilateral* amygdala volume; our examination of *unilateral* volumes arose from a data-driven decision to probe the marginal effect of secure base script knowledge on bilateral amygdala volume (*p* = 0.060) that was not included in our pre-registration. The exploratory nature and small effect size of secure base script knowledge on left amygdala volume led us to interpret these findings as preliminary. We encourage efforts to replicate these initial findings in larger samples.

It is also important to note that the observed association with amygdala volume became non-significant when adjusting for intracranial volume (eTIV), suggesting that effects may not be localized to the amygdala but may have more widespread effects; however, direct associations with eTIV and total GMV were not significant in the present study. Future research should consider effects on additional regions of interest, as well as metrics of global brain development and change in these measures over time.

What is not yet clear is exactly *how* parental secure base script knowledge is linked to child amygdala volume. Previous work shows that secure base script knowledge predicts greater parental sensitivity, positive affect, emotional support, and lower hostility and intrusiveness (Coppola et al., 2006; Huth-Bocks et al., 2014; Waters et al., 2017). In this study, however, secure base script knowledge was unrelated to mothers’ responses to child distress, and these caregiving behaviors in turn were unrelated to amygdala volume. One explanation is that the specific dimensions of caregiving assessed in present study—namely, mother-reported supportive and unsupportive responses to distress—may not be relevant mediators. Instead, observed parental sensitivity, secure base provision, autonomy support, positive affect, emotion regulation, emotional availability, sensitive touch, and engagement (vs. disengagement or neglect), as well as child factors such as attachment and emotion regulation, merit examination in future studies. Relatedly, it is possible that social desirability limited the extent to which true variation in maternal supportive and unsupportive responses to distress could be adequately captured via self-report, although ample previous work has linked this caregiving measure to maternal attachment representations (Jones et al., 2014) and observed caregiving behavior (e.g., Spinrad et al., 2007). In the present study, however, mother-reported supportive responses were highly negatively skewed, limiting variability. Some previous studies that find significant links between parenting and hippocampal volume have used observational measures of lab-based parent–child interactions (e.g., Blankenship et al., 2019); future work using observational assessments of parents’ response to distress may reveal greater variability, with meaningful links to both parent representations and child brain development. Further, future work using observational assessments with clinical samples could leverage greater variability to assess the possibility that such links manifest only at the extreme ends of the caregiving spectrum.^4^

An additional explanation for our results may be developmental timing: It is possible that caregiver responses to child distress are more salient mediators during sensitive periods for the development of attachment and for brain development, such as infancy; indeed, much of the previous work linking parental sensitivity to amygdala volume has focused on caregiving during infancy (e.g., Rifkin-Graboi et al., 2015; Bernier et al., 2019) or beginning in infancy (e.g., Tottenham et al., 2010; Lupien et al., 2011). It is also possible that the indirect effects of mothers’ representations unfold over time, such that parental secure base script knowledge predicts relative changes in caregiving, or that positive caregiving predicts relative changes in amygdala development that are best captured longitudinally, similar to findings that positive parenting predicts less relative growth of the amygdala in adolescence (Whittle et al., 2014).

Contrary to hypotheses, mothers’ attachment representations were not significantly related to hippocampal volume; only a marginal association was observed between secure base scripts and smaller hippocampal head volumes. To the extent that more secure representations forecast positive parenting (e.g., Coppola et al., 2006; Huth-Bocks et al., 2014), these findings contrast with some previous work linking positive parenting in early childhood to larger hippocampal volume among school-age children with and without depressive symptoms (e.g., Luby et al., 2012), and are instead more consistent with the null results reported in two studies with non-clinical samples in early childhood (Kok et al., 2015; Lee et al., 2019). Importantly, however, there was also no indirect effect of parent representations on hippocampal volume via mother-reported caregiving in response to distress, in part because response to distress was unrelated to hippocampal volume in the present sample. One possibility is that our focus on hippocampal structure is too coarse, as we did not have resolution to look at functional subfields. Future work could examine potential associations between parental mental representations and hippocampal subfields such as the dentate gyrus, building on previous work linking parental maltreatment with smaller left CA4-DG subfield volumes (Teicher et al., 2012). It may be that normative variations in caregiving are not enough to produce substantial change in the hippocampus. Perhaps the hippocampus is only sensitive to particularly high levels of stress. This is plausible, given the other null results in normative samples in early childhood (Kok et al., 2015; Lee et al., 2019) and the possible issue of publication bias. Or perhaps no direct link exists, though there may be indirect effects via additional caregiving and child mechanisms (e.g., observed parental sensitivity, child attachment), which warrant future research.

In follow-up exploratory analyses, mothers’ greater secure base script knowledge was associated with children’s smaller thalamus volume, smaller WMV, and larger surface area of the right pericalcarine cortex; mothers’ attachment anxiety was associated with children’s smaller thalamus volume; and maternal representations were not associated with children’s cortical thickness in any brain regions. Given their exploratory nature, we interpret them with caution.

Although not hypothesized *a priori*, findings regarding the thalamus are consistent with some previous work. Findings could be due to the fact that the thalamus and amygdala work together in stress-response circuitry, as demonstrated in research with animals (Spencer et al., 2004; Penzo et al., 2015; Wei et al., 2015) and humans (Öhman, 2005). For instance, one animal study found that the paraventricular nucleus of the thalamus (PVT) and the amygdala worked together in a circuit that established fear memory and facilitated fear responses during a fear conditioning task in mice (Penzo et al., 2015). Research in humans has demonstrated a link between children’s disorganized attachment (a correlate of insensitive care; Moran et al., 2008) and smaller gangliothalamic ovoid (comprised of the thalamus and the basal ganglia) diameter in infancy (Tharner et al., 2011). Maternal sensitivity has also been linked to smaller subcortical GMV (which includes the thalamus, as well as the caudate, putamen, and globus pallidus) in infancy (Sethna et al., 2017). It is surprising, however, that greater secure base script knowledge (indicating secure representations) and greater attachment anxiety (indicating insecure representations) predicted thalamus volume in the *same direction*. This region warrants further investigation in studies of parenting and child brain structure.

Findings regarding WMV contrast with research suggesting that smaller WMV is associated with more negative parenting experiences (Belsky and de Haan, 2011). Notably, however, the majority of these studies focus on neglected and maltreated samples (but see Sethna et al., 2017 for evidence in infants from a community sample). One possibility is that a curvilinear relation exists across the spectrum of caregiving experiences, with experiences of extreme caregiving adversity linked to abnormally small volumes across a number of brain structures (e.g., McLaughlin et al., 2019), but normative variation in caregiving quality associated with volumes in the opposite direction (although still within typical ranges, e.g., Rifkin-Graboi et al., 2015; Bernier et al., 2019). Findings regarding the right pericalcarine cortex contrast with the broader literature about structures sensitive to caregiving experiences (Belsky and de Haan, 2011; Farber et al., 2020), and given our small sample, underscore the need for future research.

That there were no significant associations between secure base script knowledge and total GMV, subcortical GMV, or intracranial volume may similarly reflect different patterns of association in non-clinical samples, compared to what we know about caregiving adversity, and/or possible non-linear associations. Although eTIV and GMV did not meet conventional thresholds for statistical significance (*p*s ∼0.10), given the exploratory nature of the work, we point out these relations as an area for future investigation with a larger sample to increase power to detect potential small effects. Another interpretation is that effects may be specific to structures involved in stress regulation and are not driven by a whole brain effect. An additional possibility is that genotype plays a role in how parental characteristics relate to child brain structure. For example, one study found that maternal acceptance was positively associated with regional GMV of the left thalamus for adolescent carriers of the *FKBP5-T* allele, but negatively associated for those without this allele (Matsudaira et al., 2019). Thus, these preliminary findings may be better understood by leveraging genetic research to examine gene by caregiving interactions.

Across our analyses, mothers’ secure base script knowledge appeared to be a more salient predictor of child brain structure, whereas maternal attachment style was largely not significant. One explanation for this difference in the predictive strength of these aspects of maternal representations is that secure base script knowledge taps scripts that reflect mothers’ own early experience receiving care, whereas self-reported adult attachment style dimensions tap experiences in current close relationships—evidenced by the relatively greater link of early sensitive care to secure base script knowledge than to attachment style (Steele et al., 2014). A substantial body of research demonstrates that narrative assessments of attachment representations such as the Adult Attachment Interview and secure base script knowledge predict child outcomes such as attachment security (e.g., Verhage et al., 2016), compared to a much smaller but growing literature on parental attachment style (Jones et al., 2015). Thus, although both attachment styles and secure base script knowledge may guide parenting behavior, parents’ scripts of how distress is met with sensitive care and co-regulation may be more important for child attachment security and in turn, the associated development of brain circuits that support the regulation of stress. Another reason may be that attachment style reflects emotion regulation strategies in close relationships broadly, whereas half of the secure base script stories refer specifically to parent–child relationships and may therefore tap into the caregiving system in ways more directly related to child brain development. More specifically, it is possible that secure base script knowledge is a better indicator of the extent to which mothers are able to effectively co-regulate their child’s distress, which in turn is linked to the development of neural circuits involved in children’s self-regulation. We cautiously suggest that mothers’ secure base script knowledge is a more relevant attachment representation than attachment style for predicting brain structure in early childhood.

### Limitations and Future Directions

Strengths of the study include its novel examination of parents’ attachment representations as a potential source of individual differences in child brain structure; its assessment of multiple conceptualizations of parents’ representations (attachment style and secure base script knowledge), including a gold standard task-based measure of secure base script knowledge; and its focus on an important period of neurodevelopment spanning preschool to school-age.

These findings, however, should be considered along with the study’s limitations. First, the small sample size and its lack of diversity limit the strength of the conclusions that can be drawn from this study, and results should be regarded as preliminary. Relatedly, we did not have adequate power to test potential moderators, such as child age, gender, race/ethnicity and exposure to discrimination, SES, or temperament. It is possible that maternal representations and/or caregiving behaviors are more strongly related to brain development among specific groups of children, such as younger children, temperamentally fearful children (who may be particularly susceptible to their caregiving environment; Belsky and Pluess, 2009), or children exposed to poverty and other stressors (i.e., reflecting a caregiving stress-buffering effect; Luby et al., 2013). Future work in larger samples across a wider age range and demographic profile could test these moderators to better characterize how factors within the child, parent, and broader bioecological context relate to individual differences in brain development. Further, effect sizes were small and research in larger samples is crucial for testing the robustness and replicability of the findings.

Second, the design was cross-sectional and correlational. Thus, although our theoretical model was based on theory and previous research, full criteria for mediation were not met because all variables were assessed at the same time point. Relatedly, we cannot establish directionality or causality; it is possible that aspects of child brain development give rise to child behaviors that elicit certain caregiving responses, or that underlying shared genetic factors inform both parental characteristics and child brain development. Thus, an important direction for future longitudinal work is to examine how parental representations, caregiving, and child brain development unfold over time (e.g., using cross-lagged analyses to establish the direction of effects), whether associations change with development, and whether sensitive periods exist for parental representations and behaviors to demonstrate links with specific brain structures. Further, experimental work could examine whether parenting interventions to shift parental attachment representations have downstream effects on child brain development. One study shows that attachment-based parenting interventions such as Attachment and Biobehavioral Catch-up (ABC; Dozier and Bernard, 2019) in infancy impact brain function in middle childhood (Valadez et al., 2020). Whether such effects are predicted by intervention-related changes in maternal representations, and whether effects extend to child brain structure, are open questions.

Third, caregiving behavior was assessed via self-report, subject to reporter bias and social desirability. Thus, it will be important to incorporate observational measures of caregiving to better understand the mechanisms by which parental representations may relate to brain structure.

Finally, although we focused on brain *structure* as a starting point for future research on this topic, it is possible that mothers’ attachment representations link more strongly to brain *function* (see Cruciani et al., 2021, for evidence of attachment-related differences in functional connectivity in the amygdala and the hippocampus). For example, research has indicated that adverse caregiving experiences can hinder the development of neural circuitry critical for stress-regulation, specifically, connectivity between the amygdala and PFC (Burghy et al., 2012; Tottenham, 2015). The amygdala is particularly sensitive to threatening stimuli, and as the PFC matures, it couples with the amygdala to modulate emotional responses to threat and reduce distress (Banks et al., 2007; Tottenham, 2015). The presence of a supportive caregiver to co-regulate a child’s distress during sensitive periods of brain development provides the scaffolding for optimal amygdala-PFC connectivity (Tottenham, 2015; Callaghan and Tottenham, 2016b). Such scaffolding can foster the amygdala-PFC coupling necessary for children’s self-regulation when a caregiver is not present (Banks et al., 2007; Dougherty et al., 2015). To the extent that mothers’ attachment representations link to caregiving behaviors that co-regulate stress (Jones et al., 2014, 2015), children’s amygdala-PFC connectivity could be a mechanism through which mothers’ attachment representations “get under the skin” and shape the development of children’s self-regulatory capacities. Studies examining links of parental representations to child brain function (e.g., amygdala–PFC connectivity, amygdala reactivity to stressors; Callaghan and Tottenham, 2016a) could be a fruitful next step.

## Conclusion

Central to attachment theory is the idea that mental representations of close relationships guide caregiving behavior and help organize the social-emotional development of the next generation (Main et al., 1985; Steele et al., 2014). The present study integrates two caregiving literatures—one demonstrating that parents’ secure mental representations support sensitive caregiving and child social-emotional adaptation (e.g., Raby et al., 2021), particularly self-regulation (e.g., Madigan et al., 2007; Waters et al., 2015), and another demonstrating that parenting behaviors meaningfully shape child brain development, particularly neural structures involved in stress regulation circuitry (e.g., the amygdala; Callaghan and Tottenham, 2016a). Our preliminary findings highlight a possible role of parents’ secure base script knowledge specifically in predicting amygdala development in early childhood; however, substantial questions remain regarding potential mechanisms, the role of developmental timing of caregiving experiences, and the generalizability of these findings to other populations (e.g., non-WEIRD samples) and ages (e.g., adolescents). Results extend findings linking mothers’ secure base script knowledge to child outcomes (e.g., Steele et al., 2014) and point to parental mental representations as an important factor to enrich research on the caregiving correlates of child brain development.

## Data Availability Statement

The datasets presented in this article are not readily available because of concerns related to participant confidentiality. Requests to access the datasets should be directed to TR, riggins@umd.edu.

## Ethics Statement

The studies involving human participants were reviewed and approved by University of Maryland Institutional Review Board. Written informed consent to participate in this study was provided by the participants’ legal guardian/next of kin.

## Author Contributions

MF: coordinating the project, generating research questions, conducting statistical analyses, writing, and editing. JS: generating research questions, coding the Attachment Script Assessment, writing, and editing. MS: coding the Attachment Script Assessment, writing, editing, and generating research questions. TA: recruiting participants, collecting data, generating research questions, consulting, processing and analyzing MRI data, assisting with statistical analyses, and editing. JC: generating research questions, consulting, writing, and editing. TR: designing and overseeing Study 1 and Study 2, recruiting participants, collecting data, generating research questions, consulting, writing, and editing. All authors: contributed to the article and approved the submitted version.

## Author Disclaimer

The content is solely the responsibility of the authors and does not necessarily represent the official views of the National Institutes of Health.

## Conflict of Interest

The authors declare that the research was conducted in the absence of any commercial or financial relationships that could be construed as a potential conflict of interest.

## Publisher’s Note

All claims expressed in this article are solely those of the authors and do not necessarily represent those of their affiliated organizations, or those of the publisher, the editors and the reviewers. Any product that may be evaluated in this article, or claim that may be made by its manufacturer, is not guaranteed or endorsed by the publisher.

## Footnotes

1
https://osf.io/eckj3

2
http://surfer.nmr.mgh.harvard.edu/

3
nitrc.org/projects/segadapter

4 We conducted an exploratory *post hoc* analysis to examine whether links between mothers’ responses to child distress and children’s brain structures differed based on mothers’ levels of supportive and unsupportive responses to distress. We used median splits for supportive and unsupportive responses to distress to create groups that were high vs. low for each. When examining links within each group separately, there were still no significant associations between responses to distress and hippocampal or amygdala volumes (all *p*s > 0.05).
